# Analysis of Immunization, Adverse Events, and Efficacy of a Fourth Dose of BNT162b2 Vaccine in Health Workers in Mexico, a Pilot Study

**DOI:** 10.3390/vaccines10071139

**Published:** 2022-07-17

**Authors:** Maria Elena Romero-Ibarguengoitia, Arnulfo González-Cantú, Diego Rivera-Salinas, Yodira Guadalupe Hernández-Ruíz, Ana Gabriela Armendariz-Vázquez, Irene Antonieta Barco-Flores, Rosalinda González-Facio, Miguel Ángel Sanz-Sánchez

**Affiliations:** 1 Department of Research, Hospital Clínica Nova de Monterrey, San Nicolás de los Garza 66450, Nuevo León, Mexico; c.argoca@novaservicios.com.mx (A.G.-C.); diego.rivera@udem.edu (D.R.-S.); yodira.hernandez@udem.edu (Y.G.H.-R.); ana.armendariz@udem.edu (A.G.A.-V.); c.ibarco@novaservicios.com.mx (I.A.B.-F.); rgonzalezf@novaservicios.com.mx (R.G.-F.); msanzs@novaservicios.com.mx (M.Á.S.-S.); 2 Vicerrectoría de Ciencias de la Salud, Escuela de Medicina, Universidad de Monterrey, San Pedro Garza García 66238, Nuevo León, Mexico

**Keywords:** coronavirus, COVID-19, vaccination, immunization, adverse effects, antibodies, fourth dose

## Abstract

There is scarce information on seroconversion and adverse events after immunization (AEFI) with the fourth dose of BNT162b2. Our aim was to correlate the magnitude of the antibody response to this vaccination regimen in terms of clinical conditions and AEFI. This was an observational pilot study in which SARS-CoV-2 S1–S2 IgG antibodies titers were measured 21–28 days after the first and second dose, three months after the second dose, 1–7 and 21–28 days after the third dose, before the fourth dose, and 21–28 days after the fourth dose. We recruited 112 subjects in a hospital in Mexico, 74% women, with an average age of 43 (SD 9) years. After the first dose, subjects had a median IgG AU/mL (IQR) of 122 (1904) that increased to 1875 (2095), 3020 (2330), and 4230 (3393) 21–28 days after the second, third, and fourth doses, respectively (*p* < 0.01). The number (%) who experienced any AEFI between the first and fourth doses was 90 (80.4), 89 (79), 65 (58), and 69 (61.5), respectively (*p* < 0.001). After the fourth dose, the most frequent of AEFI was pain at the injection site (87%). There was a correlation between AEFI and gender after the fourth dose, as well as with antibody levels (*p* < 0.05). During the Omicron outbreak, six (5.3%) had mild COVID-19 for 8–28 days after the fourth dose. The median increase in S1/S2 IgG was 30.8-fold after the fourth BNT162b2 dose when compared with the first dose and caused mild AEFI.

## 1. Introduction

The SARS-CoV-2 worldwide pandemic has burdened all health systems due to the agent’s high contagiousness and unpredictable outcome. As of June 2022, globally, SARS-CoV-2 has caused a total of 530,266,292 confirmed cases and 6,299,364 reported deaths according to the World Health Organization (WHO). In Mexico, as of June 2022, 5,791,282 confirmed cases and 325,017 deaths have been reported [1]. This agent has been proven to cause multiple waves of disease, with more transmissible variants leading to long-term sequelae and perpetuating its unbridled impact on the economy and healthcare system [2].

Currently, 122 vaccines to prevent SARS-CoV-2 disease and/or severe cases are being tested, of which 49 are at the final step in clinical trials [3,4]. As of June 2022, worldwide, 11,832,555,347 vaccine doses have been administered. In Mexico, vaccination against SARS-CoV-2 began in late December 2020. Currently, 86,858,994 Mexicans have at least one vaccination dose, 79,947,470 are fully vaccinated with a complete scheme, and 53,001,421 have received at least one booster [1].

Based on the mechanism of action, vaccines can be clustered in four groups: (1) mRNA vaccines where genetically engineered, modified RNA is used to produce the spike protein that safely prompts an immune response. (2) Viral vector (adenovirus) vaccines that contain a genetically engineered virus that can generate coronavirus proteins and a further immune response without causing the disease. (3) Inactivated virus vaccines that inoculate a form of the virus that has been inactivated or weakened and is incapable of causing disease while still generating an immune response. (4) Protein subunits vaccines that use harmless fragments of proteins or protein shells that mimic SARS-CoV-2 to safely provoke an immune response [5]. PfizerBioNTech (Pfizer Inc, New York, NY, USA, and BioNTech SE, Mainz, Germany) was one of the first laboratories to develop a mRNA vaccine against SARS-CoV-2, and it was named BNT162b2 [6,7].

The duration of immunity generated by vaccination is of primary interest. Previous studies showed a decline in antibody titers three to six months after the complete vaccination regimen. For example, Khoury et al. showed in their study of health workers that a drop in antibody titers occurred four months after the complete vaccination regimen, reaching only 6.3% of the maximal titer [8]. Other studies in subjects with lung transplant, primary immunodeficiencies, and inborn errors of immunity showed that some of them were unable to develop humoral and cellular response after vaccination [9,10,11].

As of today, the Centers for Disease Control and Prevention (CDC) has approved the application of a booster shot with BNT162b2 or mRNA-1273 (Moderna TX, Inc., Cambridge, MA, USA; Rovi Pharma Industrial Services S.A., Madrid, Spain) at least 5 months after the original vaccination scheme in all individuals 12 years and older and 18 years and older, respectively [12]. Furthermore, in immunocompromised patients, the CDC recommends a three-dose vaccination scheme with an additional dose 3 months after the third dose [13].

At the beginning of 2022, Pfizer and BioNTech applied to the Food and Drug Administration (FDA) for the approval of a fourth dose of BNT162b2 in order to enhance the vaccine’s efficacy in reducing the rates of infection and severe illness, especially in adults 65 years and older [14]. The application of a fourth BNT162b2 dose in non-immunocompromised patients requires further study. Therefore, our study aimed to correlate the magnitude of the antibody response to vaccination in terms of previous clinical conditions and the development of adverse events following immunization (AEFI) with a fourth dose of BNT162b2 mRNA in health workers [15,16]. Our secondary aim was to describe the incidence of SARS-CoV-2 infection.

## 2. Materials and Methods

This was a prospective observational pilot study that followed healthcare workers who received a complete vaccination scheme with the BNT162b2 vaccine in 2021, plus a third and fourth BNT162b2 booster 4 to 6 months after each previous dose. This study was conducted in a hospital in Northern Mexico. The study followed STROBE guidelines [17]. Our study was approved by the local Institutional Review Board (Ref. 26022021-CN-1e-CI) and was conducted as per the Code of Ethics of the World Medical Association (Declaration of Helsinki) for experiments involving humans.

The inclusion criteria were: being a healthcare professional of either gender and any age who consented to participate; a complete BNT162b2 national vaccination regimen where a third BNT162b2 dose was received 4–6 months after the original vaccination scheme and a BNT162b2 booster 4–6 months following the third dose. Exclusion criteria were: subjects vaccinated prior to the study entry; a heterologous vaccination regimen; and the application of any booster with any COVID-19 vaccine different from BNT162b2.

Initially, the participants were recruited when the health system initiated the vaccination campaigns. We invited healthcare workers who intended to complete the BNT162b2 scheme to participate in the study. Inclusion/exclusion criteria were applied. Every participant signed an informed consent form in which the study was explained. A plasma blood sample was collected at every follow up, and a questionnaire was applied. Concern over SARS-CoV-2 variants (Delta and Omicron) led participants to inform us when they received a third and fourth BNT162b2 booster on their own (all doses were 0.3 mL). Since our research protocol was approved after participants had received the first dose, blood samples were collected 21–28 days after the first and second BNT162b2 dose (T1 and T2, respectively), 3 months after the second dose (T3), 1–7 days after the third dose (T4), 21–28 days following the third BNT162b2 dose (T5), 3 months after the third dose (T6), prior to the application of the fourth dose (T7), and 21–28 days following the fourth BNT162b2 dose (T8).

The first questionnaire included questions on the patient’s medical history and whether they had developed a previous SARS-CoV-2 infection. The information in the follow-up questionnaires applied 21–28 days after the four BNT162b2 doses included the development of AEFI and SARS-CoV-2 infections after each dose.

### 2.1. Primary Outcome and IgG Determination

Our main outcome was the detection of the humoral response elicited by vaccination through the measurement of specific antibodies against SARS-CoV-2 and correlating the results with previous SARS-CoV-2 infection, AEFI, and clinical conditions. Specific anti-S1-S2 IgG antibodies against SARS-CoV-2 were measured by laboratory personnel using a chemiluminescence immunoassay (CLIA) by DiaSorin. This assay has a sensitivity of 97.4% (95% CI, 86.8–99.5) and a specificity of 98.5% (95% CI, 97.5–99.2). The results were interpreted as follows: <12.0 AU/mL as a negative result; 12.0 to 15.0 AU/mL as indeterminate; and >15.0 AU/mL as a positive result.

The variables we analyzed were gender, age, previous medical history (e.g., hypertension, type 2 diabetes mellitus, asthma, obesity, obstructive pulmonary disease, any heart condition, liver conditions, cancer, smoking, autoimmune disease), and previous confirmed SARS-CoV-2 infection (through nasal swab or serologic tests). The AEFI reported for any BNT162b2 dose (i.e., pain at the injection site, fever (>37.5 °C), headache, adenopathy) were also analyzed.

### 2.2. Statistical Methods

The sample size included all the healthcare workers that self-reported receiving a third and fourth dose of BNT162b2. The researchers reviewed the quality control and anonymization of the database. Normality assumption was evaluated with the Shapiro–Wilk or Kolmogorov tests. Log10 transformations were used when appropriate. We used descriptive statistics for the analysis, such as frequencies, percentages, medians, interquartile ranges, means, and standard deviations. We performed a Friedman test to compare the anti-S1 and -S2 antibody titers over time and the Mann–Whitney U test to compare antibody levels between subjects previously exposed to SARS-CoV-2. Cochran’s Q test was used to compare AEFI between vaccine doses.

We developed a mixed model in which the dependent variable was the antibody titers. The reference group was the anti-S1-S2 IgG antibody titers 21–28 days following the first BNT162b2 dose. We considered an individual variation as a random effect, and the fixed effects were the timing of each anti-S1-S2 antibody determination, gender, and age. Finally, we computed Poisson generalized linear models in which the number of AEFI after the fourth dose was the outcome variable. Included regressors were gender, SARS-CoV-2 history, and the number of times the antibody was measured (see Method section).

Completely missing at random values (<30%) were imputed using multiple imputations through regression models. SPSS version 25 and R v. 4.0.4 were the statistical programs used to analyze data. The analysis was two-tailed, and a *p*-value < 0.05 was considered statistically significant.

## 3. Results

One hundred and twelve (112) recruited subjects were vaccinated with a fourth BNT162b2 dose, and 83 (74%) were women. The group’s mean (SD) age was 43 (9) years. Most of the participants were nurses (n = 64, 57%) and medical providers (n = 20, 17%). The most commonly reported comorbidity was obesity (n = 32, 28.6%), followed by hypertension (n = 12, 10.7%) and dyslipidemia (n = 9, 8.0%). Seven subjects (6.3%) were active smokers, and two (1.8%) referred the use of immunosuppressive therapy. Supplementary Material Table S1 shows the medical history reported by the participants.

### 3.1. Antibody Titers

The anti-S1-S2 IgG antibody titers against SARS-CoV-2 showed significant changes over time after applying BNT162b2 booster doses. Median (IQR) antibody titers (AU/mL) after the first dose was 122 (1904.5); 21–28 days after second dose, 1875 (2095.0); three months after two doses, 300 (544.9); 1–7 days after the third dose, 489 (1055.8); 21–28 days after the third dose, 3020 (2330.0); three months after the third dose, 1035 (920.0); prior to the fourth dose, 598 (728.5); and 21–28 days after the fourth dose, 4230 (3393.0).

When comparing the antibody titers 21–28 days after the second, third, and fourth doses versus those reported after the first dose, there was a 10.66, 21.03, and 30.81 median- fold increase, respectively (*p* < 0.001). Finally, there was a statistical difference between antibody levels 21–28 days after the third and fourth dose (median difference of 1210 AU/mL, *p* < 0.001).

Three months after the second and third dose, there was a decrease of 84% and 65.7%, respectively. Before the fourth dose, 155 (22) days after the third dose, there was a decrease of 80.2%.

**Figure 1:**
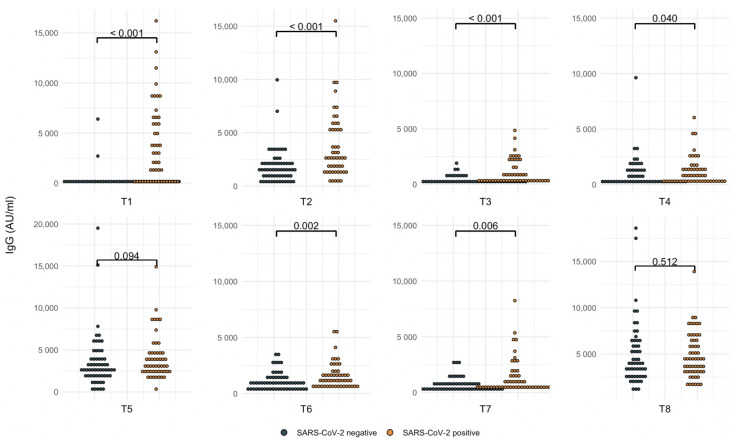
Anti-S1-S2 IgG antibody titers according to SARS-CoV-2 infection history.

Subjects were divided according to SARS-CoV-2 positivity at different time points. Antibody titers were significantly higher in cases with a positive history of SARS-CoV-2 infection (*p* < 0.05), except in the titers detected 21–28 days after the third and fourth doses, where, independently of SARS-CoV-2 infection, the titers were the same (*p* = 0.09 and *p* = 0.5, respectively). See Table 1 for antibody comparisons over time, the median-fold increase, and SARS-CoV-2 infections over time. Figure 1 represents the anti-S1-S2 IgG antibody titers.

Figure 1 shows the cumulative cases according to positive or negative SARS-CoV-2 infection, n = 112. T1: 21–28 days after the first BNT162b2 dose (SARS-CoV-2 positive = 49). T2: 21–28 days after the second dose (SARS-CoV-2 positive = 50). T3: 3 months after the second dose (SARS-CoV-2 positive = 50). T4: 1–7 days after the third dose (SARS-CoV-2 positive = 52). T5: 21–28 days after the third dose (SARS-CoV-2 positive = 52), T6: 3 months after the third dose (SARS-CoV-2 positive = 52). T7: before the fourth dose (SARS-CoV-2 positive = 52). T8: 21–28 days after the fourth dose (SARS-CoV-2 positive = 58).

We constructed a mixed model in which the anti-S1-S2 IgG antibody titers represented the dependent variable. The reference group for comparison was the antibody titers 21–28 days after the first dose. The antibody titers were analyzed in log10. This model found a significant positive effect in subjects with a SARS-CoV-2 infection history (β = 0.32, *p* < 0.001). There was a positive effect after the application of the second, third, and fourth BNT162b2 doses (β = 0.79, β = 1.0, and β = 1.2, respectively) (*p* < 0.001), in which the greatest effect was secondary to the administration of the fourth dose.

There was a decline in antibody titers at the three-month follow up after the second and third doses. However, the titers were maintained above the antibody levels after the first BNT162b2 dose. The model did not show an effect of age or sex. Table 2 shows the mixed model of the anti-S1-S2 IgG antibody titers.

### 3.2. Adverse Events Following Immunization (AEFI)

We evaluated the AEFI reported after each of the four doses. Fewer AEFI were reported after the third and fourth doses (first, n = 90, 80.4%; second, n = 89, 79.0%; third, n = 65, 58.0%; and fourth dose, n = 69, 61.6%). The time to the development of the AEFI after the first and second doses was most frequently in the first four hours after the vaccine (n = 73, 81.1%, and n = 41, 46.1%, respectively) (*p* < 0.001); after the third and fourth doses, AEFI appeared 5 to 24 h after vaccination (n = 38, 58.5%, and n = 36, 51.4%, respectively) (*p* < 0.001).

The most common of the AEFI from the four BNT162b2 vaccines was pain at the injection site (n = 80, 88.9%; n = 80, 89.0%; n = 52, 80.0%; and n = 61, 87.0%, respectively), followed by headache (n = 34, 37.8%; n = 38, 42.7%; n = 26, 40.0%; and n = 33, 47.1%, respectively) and fatigue (n = 22, 19.6%; n = 39, 43.8%; n = 27, 41.5%; and n = 29, 41.4%, respectively). When comparing AEFI after each dose, adenopathy was proportionally more frequent after the fourth dose (*p* < 0.001) and fever after the third (*p* = 0.036).

Severity in the two-dose scheme was reported as very mild (first, 60, 78.9%, and second dose, 42, 47.7%) (*p* = 0.01). However, the severity was mild after the third and fourth shots (24, 37.5%, and 36, 51.4%, respectively). See Table 3.

We computed Poisson generalized linear models to evaluate the predictors of the development of any AEFI after the fourth dose of BNT162b2. Women correlated with more episodes of AEFI (log IRR −0.59, *p* < 0.001). In addition, there was a small effect on antibody levels (log IRR 0.000029). A marginal effect was observed in terms of SARS-CoV-2 history (log IRR 0.16, *p* = 0.059). See Table 4.

### 3.3. SARS-CoV-2 Infection

We considered a subject to have a positive SARS-CoV-2 infection history when they had previously received a confirmatory swab or serologic test. Throughout follow up, 58 (51.8%) subjects reported at least one SARS-CoV-2 infection, of which 43 (38.4%) were infected once, 14 (12.5%) twice, and 1 (0.9%) three times.

Before the vaccination scheme, 49 (43.8%) subjects reported a SARS-CoV-2 infection with the original variant, 47 (95%) of which received ambulatory treatment, 2 (4%) were hospitalized, and 1 (2%) was admitted to an Intensive Care Unit (ICU). Between the first and second dose, one (0.9%) participant was infected with the original variant. Between the second and third dose, one (0.9%) subject was infected with the Alpha variant. Between the third and fourth dose, six (5.4%) cases were infected with the Delta variant, and all were infected at least eight days after receiving the vaccine and two five months after this dose. After the fourth dose during the Omicron wave, 12 (10.7%) were infected; six (5.3%) were infected 8–28 days after the fourth dose. All SARS-CoV-2 infection cases reported after administration of the first BNT162b2 dose received ambulatory treatment.

## 4. Discussion

This pilot study analyzed the effect of a fourth BNT162b2 dose in terms of its immunogenicity by measuring anti-S1-S2 IgG antibodies. Additionally, AEFI and SARS-CoV-2 infections were addressed. The major strength of our study is the close follow up of the population.

The fourth dose led to a median 30.8-fold increase in antibody titers in comparison with the first dose. There was a positive effect in antibody titers when subjects had been previously exposed to SARS-CoV-2. This effect faded when healthcare workers received the third and fourth doses. As previously stated, three to six months following the second BNT162b2 dose, antibodies tended to decrease over time, and, three months after the third dose, the same effect was observed.

Regev-Yochay et al. conducted a study in which they found a 9–10-fold increase in antibody titers after a fourth dose, but they found no substantial difference between the third and fourth dose results. They concluded that the fourth dose’s effect might peak the antibody titers rather than increase immunity [18]. In our study, we found a median 30.8-fold increase in antibodies after the fourth dose compared to the first dose, and, in comparison, with the increase 4–6 months after the third dose, we found a seven-fold increase.

The difference in the increase in antibodies between studies could be related to the method used for antibody measurement. In addition, we had higher mean level of antibody titers during follow up, which could have been due to the inclusion of patients with a positive SARS-CoV-2 history. Finally, when comparing the antibody levels after the third and fourth dose, we found a statistical difference, with a 1.4-fold increase.

The fourth BNT162b2 vaccine was safe because there were no severe adverse effects after vaccination. Limited mild, local, and systemic AEFI were reported following the fourth dose, but no major complications. After the four doses, the most frequently reported AEFI were pain at the injection site, headache, and fatigue. We found that the female gender and the antibody titers correlated with the development of AEFI. Regev-Yochay et al. reported no serious adverse events and no hospital admissions related to vaccination, concluding that the fourth BNT162b2 dose was as safe as the previous vaccines [18]. Although the most common AEFI in their study were fatigue, myalgia, and headache, these were low grade and self-limiting, as in our study.

The previous study reported that the cumulative incidence of SARS-CoV-2 infection with Omicron over the 8–29-day period after administration of the BNT162b2 vaccine was 18.3% compared to 25.3% (95% CI: 18.5 to 31.5%) among controls [18]. Our study reported a rate of 10.7% but 5.3% eight days after the fourth dose: a lower infection rate. Additionally, when comparing the infection rate at least eight days after the third and fourth dose, there was a similar rate of SARS-CoV-2 infection, even though it is well known that the Omicron variant is more contagious and has a higher infection rate [19,20].

Recently, the FDA and CDC recommended a second booster in subjects above the age of 50 or who are immunocompromised; the fourth dose is recommended to be given at least four months after the third booster [21,22]. Other studies suggested that a fourth dose has low efficacy in the prevention of mild or asymptomatic Omicron infections and that the infectious potential still exists, so there is an urgency for the development of next-generation vaccines [23,24]. We agree with previous publications, but, until a new generation of vaccines is available, a fourth dose regimen could be an option for elderly and immunocompromised patients. In younger and healthy subjects, our study showed it could prevent severe cases of SARS-CoV-2 infection and decrease hospital burden.

As to our study’s limitations, it was of an observational nature, so we lacked randomization and a control group; however, patients were followed closely. A larger sample size would be of interest to test other predictors of antibody change; however, the changes in antibody titers between doses were so different that we were able to achieve sufficient statistical power for the inferences addressed in this manuscript. This study did not include baseline antibody values since all participants were included in the protocol after having received the first dose. This study included young healthcare workers. It is difficult to conclude what this booster’s efficacy would be in the elderly and in children. We only analyzed two subjects with immunosuppression, so a greater sample size is required to reach valid conclusions in this subgroup of subjects. However, we believe that this manuscript is valuable since new SARS-CoV-2 variants are emerging, and there is scant information on a fourth dose that could help prevent infection or severe COVID-19 cases.

## 5. Conclusions

In conclusion, the fourth dose of BNT162b2mRNA triggered a humoral response by increasing by 30.8 times the S1/S2 IgG in comparison with one dose, and it had mild adverse events. Women had more AEFI after the fourth dose, and there was a small effect related to the antibody level. Although the Omicron variant is contagious, we found a similar infection rate to the Delta variant after subjects received the fourth dose. All the infected cases had mild symptoms and were treated as outpatients. Previous SARS-CoV-2 infection did not reflect long-lasting immunity; we suggest and encourage vaccination as a strategy to prevent the disease. The third and fourth doses of BNT162b2 are safe and effective.

## Figures and Tables

**Table 1:** Follow up of anti-S1-S2 IgG antibody titers according to SARS-CoV-2 infection history.

Time Points	Total (n = 112) (AU/mL)	Median-Fold Increase ^1^ (n = 112)	Negative SARS-CoV-2 Infection (AU/mL) Before Vaccination (n = 63)	Median-Fold Increase Negative SARS-CoV-2 Infection	Positive SARS-CoV-2 Infection (AU/mL) Before Vaccination (n = 49)	Median-Fold Increase Positive SARS-CoV-2 Infection	*p*-Value
21–28 days after first dose (T1)	122 (1837.8)		97.6 (68.6) n = 63		2220 (6005) n = 49		<0.001
21–28 days after second dose (T2)	1875 (2065)	10.66	1495 (1430) n = 62	14.1	2680 (3920) n = 50	1.52	<0.001
Three months after second dose (T3)	300 (540.2)	1.98	217 (236) n = 62	2.58	452 (1355) n = 50	0.36	<0.001
1–7 days after third dose (T4)	489 (1019.2)	2.22	416 (988) n = 60	3.85	740 (1245) n = 52	0.46	0.040
21–28 days after third dose (T5)	3020 (2210)	21.03	2805 (1765) n = 60	32.2	3256 (2403) n = 52	1.72	0.094
Three months after third dose (T6)	1035 (914.6)	6.06	853 (813) n = 60	8.5	1285 (1052) n = 52	0.62	0.002
Prior to the application of fourth dose (T7)	598 (707.5)	2.98	448 (492) n = 60	4.85	693 (1270) n = 52	0.55	0.006
21–28 days after fourth dose (T8)	4230 (3337.5)	30.81	4080 (3638) n = 54	40	4455 (3302) n = 58	2.4	0.512
*p*-value	<0.001	<0.001	<0.001		<0.001		

SARS-CoV-2 infection is presented as cumulative case count. Some subjects could have had SARS-CoV-2 more than once. Data are presented as medians and interquartile ranges. The Friedman test was performed for comparison between time points. The Mann–Whitney U test was applied for comparisons between individuals with a positive and negative SARS-CoV-2 infection. A *p* < 0.05 was considered statistically significant. ^1^ The increase throughout follow up was compared with the antibody titers determined 21–28 days after the first dose.

**Table 2:** Mixed model of the anti-S1 and -S2 antibody titers against SARS-CoV-2.

	Estimate (β)	Std. Error	95% CI	*p*-Value
Intercept	0.27			
Age	0.00	0.003	−0.01, 0.00	0.15
Sex	−0.05	0.065	−0.18, 0.07	0.4
SARS-CoV-2 infection before vaccination	0.32	0.057	0.21, 0.43	<0.001
21–28 days after second dose	0.79	0.047	0.70, 0.88	<0.001
Three months after second dose	0.10	0.047	0.00, 0.19	0.044
1–7 days after third dose	0.27	0.047	0.18, 0.36	<0.001
21–28 days after third dose	1.0	0.047	0.91, 1.1	<0.001
Three months after third dose	0.55	0.047	0.46, 0.64	<0.001
Prior to fourth dose	0.35	0.047	0.25, 0.44	<0.001
21–28 days after fourth dose	1.2	0.047	1.1, 1.3	<0.001

Mixed model in which the anti-S1 and -S2 antibody titers against SARS-CoV-2 represented the dependent variable. Antibody titers are analyzed as log10. Antibody titers 21–28 days after the first BNT162b2 dose were the reference group. A *p*-value < 0.05 was considered statistically significant.

**Table 3:** Adverse events following immunization after each administered BNT162b2 dose.

AEFI (n = 112)	First Dose (%)	Second Dose (%)	Third Dose (%)	Fourth Dose (%)	*p*-Value
Presence of any AE	90 (80.4)	89 (79.0)	65 (58.0)	69 (61.6)	<0.001
Time of appearance					
First 4 h after	73 (81.1)	41 (46.1)	8 (13.8)	22 (31.4)	<0.001
5 to 24 h after	5 (4.5)	24 (27.0)	38 (58.5)	36 (51.4)	<0.001
2 to 3 days after	12 (13.3)	22 (24.7)	17 (26.2)	12 (17.1)	<0.001
4 to 7 days after	0	1 (1.1)	1 (1.5)	0	
7 to 10 days after	0	1 (1.1)	0	0	
Symptoms					
Pain at injection site	80 (88.9)	80 (89.0)	52 (80.0)	61 (87.0)	0.838
Adenopathy	1 (1.1)	1 (1.1)	7 (10.8)	11 (15.7)	<0.001
Fever (>38 °C)	4 (4.4)	1 (1.1)	9 (13.8)	8 (11.4)	0.036
Arthralgias	5 (5.6)	25 (28.1)	14 (21.5)	16 (22.9)	0.019
Headache	34 (37.8)	38 (42.7)	26 (40.0)	33 (47.1)	0.303
Fatigue	22 (19.6)	39 (43.8)	27 (41.5)	29 (41.4)	0.110
Myalgias	7 (7.8)	26 (29.2)	16 (24.6)	15 (21.4)	0.135
Local edema or erythema	5 (5.6)	14 (15.7)	6 (9.2)	8 (11.4)	0.432
Low-grade fever (37.5–37.9 °C)	4 (4.4)	10 (11.2)	6 (9.2)	12 (17.1)	0.188
Nausea	4 (4.4)	6 (6.7)	3 (4.6)	4 (5.7)	0.392
Palpitations	3 (3.3)	2 (2.2)	3 (4.6)	2 (2.9)	0.836
Nasal congestion	3 (3.3)	10 (11.2)	3 (4.6)	4 (10)	0.095
Diarrhea	2 (2.2)	4 (4.5)	2 (3.1)	1(1.4)	0.392
Pruritus	2 (2.2)	5 (5.6)	1 (1.5)	3 (4.3)	0.682
Ocular pain	2 (2.2)	0	0	0	-
Insomnia	2 (2.2)	1 (1.1)	2 (3.1)	0	0.733
Localized skin rash	1 (0.9)	2 (2.2)	0	1 (1.4)	0.572
Vomiting	1 (1.1)	1 (1.1)	0	2 (2.9)	0.572
Back pain	1 (1.1)	0	0	0	-
Achromatopsia	1 (1.1)	0	0	0	-
Chest pain	0	4 (4.4)	1 (1.5)	2 (2.9)	0.392
Hypotension	0	0	1 (1.5)	1 (1.4)	-
Diffuse skin rash	0	0	1 (1.5)	0	-
Systemic edema	0	1 (1.1)	0	0	-
Severity					
Very mild	60 (78.9)	42 (47.7)	21 (32.8)	20 (28.6)	0.01
Mild	6 (7.9)	27 (30.7)	24 (37.5)	36 (51.4)	0.01
Moderate	10 (13.2)	18 (20.5)	17 (26.6)	12 (17.1)	0.01
Severe	0	1 (1.1)	1 (1.6)	2 (2.9)	-
Very severe	0	0	1 (1.6)	0	-

Data are presented as frequencies and percentages. Cochran’s Q test was performed for comparisons. A *p*-value < 0.05 was considered statistically significant. AEFI: adverse events following immunization.

**Table 4:** Poisson generalized linear models of factors related to adverse events following immunization.

Variable	Log (IRR)	95% CI	*p*-Value
Gender	−0.59	−0.82, −0.37	<0.001
SARS-CoV-2 infection before vaccination	0.16	−0.01, 0.33	0.059
Antibody titers	0.0000292	0.0000003.00005	0.048

IRR: incidence rate ratio, CI: confidence interval. Poisson generalized linear regression was performed to analyze the factors related to adverse events following immunization. A *p*-value < 0.05 was considered statistically significant.

## Data Availability

Data are available upon reasonable request to the authors.

## Supplementary Materials

The following are available online at https://www.mdpi.com/article/10.3390/vaccines10071139/s1, Table S1: Medical History.
