# Supplementary material for: Analysis of Immunization, Adverse Events, and Efficacy of a Fourth Dose of BNT162b2 Vaccine in Health Workers in Mexico, a Pilot Study
Source: Vaccines (Basel). 2022 Jul 17;10(7):1139. doi: 10.3390/vaccines10071139 (PMC9318868; doi:10.3390/vaccines10071139)
Supplement: Supplementary file 1 [file vaccines-10-01139-s001.zip › vaccines-1733413-supplementary.pdf]

Supplementary Material

Table S1. Medical History.

Medical history (n = 112)	Frequency (%)
Obesity	32 (28.6)
Hypertension	12 (10.7)
Dyslipidemia	9 (8.0)
Prediabetes	8 (7.1)
Hypothyroidism	7 (6.3)
Smoking	7 (6.3)
Type 2 Diabetes Mellitus	6 (5.4)
Non-alcoholic fatty liver disease	4 (3.6)
Asthma	3 (2.7)
Rheumatoid arthritis	2 (1.8)
Use of immunosuppressive therapy	2 (1.8)
Atrial fibrillation	1 (0.9)
Heart failure	1 (0.9)
Coronary heart disease	1 (0.9)
Stroke	1 (0.9)
Gout	1 (0.9)
Pregnancy	1 (0.9)

Data are presented in frequencies and percentages.
